# Insights and Lessons from Chilean Salmon Aquaculture on Antimicrobial Use

**DOI:** 10.3390/antibiotics14121177

**Published:** 2025-11-21

**Authors:** Daniela R. Farias, Rolando Ibarra, Felipe Tucca, Alexander Jaramillo-Torres, Javiera Cornejo, Pablo Ibieta, Fernando O. Mardones, Ruben Avendaño-Herrera

**Affiliations:** 1 Monterey Bay Aquarium, Global Oceans Conservation Program, 886 Cannery Row, Monterey, CA 93940, USA; 2 Wavet Research, Gramado 1100, Puerto Varas 5550000, Chile; 3 Instituto Tecnológico del Salmón, INTESAL, Puerto Varas 5550000, Chile; 4 FAVET, Facultad de Ciencias Veterinarias y Pecuarias, Universidad de Chile, Santiago 8820808, Chile; 5 Center for Antimicrobial Stewardship in Aquaculture (CASA), Santiago 8820808, Chile; 6 TEKBios, Astillero Hijuela 3 Km 8, Maullin 5580000, Chile; 7 PINCOY Project, Puerto Montt 5480000, Chile; 8 Global Academy of Agriculture and Food Systems, Royal (Dick) School of Veterinary Studies, The Roslin Institute, Easter Bush Campus, Midlothian EH25 9RG, UK; fmardone@ed.ac.uk; 9 School of Veterinary Medicine, Pontificia Universidad Católica de Chile, Macul, Santiago 7780366, Chile; 10 Laboratorio de Patología de Organismos Acuáticos y Biotecnología Acuícola, Facultad de Ciencias de la Vida, Universidad Andrés Bello, Viña del Mar 2340000, Chile; 11 Interdisciplinary Center for Aquaculture Research (INCAR), Viña del Mar 2340000, Chile; 12 Centro de Investigación Marina Quintay (CIMARQ), Universidad Andrés Bello, Quintay 2480055, Chile

**Keywords:** aquaculture, antimicrobial resistance, disease management, one health

## Abstract

Background: Chilean salmon aquaculture, a sector crucial for global food security, faces persistent challenges from bacterial pathogens, particularly *Piscirickettsia salmonis*, resulting in substantial antimicrobial use (351.1 tons in 2024). Objective and methods: To address this issue, the “Sustainable Management of Aquaculture Bacterial Diseases” workshop convened 27 experts to develop a roadmap for prudent antimicrobial stewardship, with the goal of informing public policies and industry best practices. Discussions focused on four critical areas. Results: Antimicrobial Resistance Prevention recognized aquaculture’s dual role in antimicrobial resistance development, underscoring its ecological dimension, the need for international collaboration, proactive policy design, and the establishment of comprehensive “One Health” surveillance systems guided by expert committees. Communication and Education identified critical gaps in specialized veterinary aquaculture training and public outreach, advocating for interdisciplinary teams and clear communication strategies, with a projected timeline of a decade for effective implementation. Therapeutic Alternatives acknowledged current limitations of vaccines against *P. salmonis* but underscored promising Chilean innovations, including the use of plant extracts, gut-microbiota modulation, and passive immunotherapy, as well as the importance of early intervention. Finally, environmental impact discussions revealed data deficiencies in aquatic ecosystems, emphasizing the need for advanced molecular tools, adaptive regulatory frameworks, and harmonized requirements for environmental risk-assessment procedures. Conclusions: The workshop’s findings provide a vital framework for advancing sustainable antimicrobial use within the Chilean salmon industry as a case study. The insights and lessons derived from this sector can significantly contribute to global aquaculture’s transition toward sustainability, providing a solid foundation for developing a comprehensive roadmap and universally applicable recommendations for stakeholders across aquaculture and other animal-farming industries worldwide. This roadmap, with its essential components, is designed to enhance the understanding of AMU in farmed salmon through a One Health approach, incorporating global guidance for all aquaculture stakeholders.

## 1. Introduction

Salmon farming is the primary production activity in Chile’s aquaculture sector, with a total output of 1,035,307 harvested tons in 2024 [1]. Despite its significant contribution to the national economy and global food security, the sector faces critical challenges in controlling bacterial diseases, which have posed a threat to sustainability for decades. Although Chile’s salmon aquaculture reports the highest fish survival rates among the top salmon-producing nations, it remains the most affected by infectious diseases, with annual revenue losses estimated at over US$700 million [2,3]. The most significant bacterial disease is caused by *Piscirickettsia salmonis*, the etiological agent of piscirickettsiosis, commonly known as Salmonid Rickettsial Septicemia (SRS), a condition that causes severe economic losses for the Chilean salmon industry [4,5,6,7,8]. Recently, the Chilean National Fisheries and Aquaculture Service (SERNAPESCA, Spanish acronym) reported a total antimicrobial (AM) use of 351.1 tons, with 97.9% of treatments administered during the marine grow-out phase to treat SRS [1]. Despite the research on piscirickettsiosis, there are still no effective vaccines available to control this disease [9]. Further efforts are therefore needed to close research gaps, mitigate the impact of bacterial diseases, and improve fish health management [6].

While antimicrobial use (AMU) remains essential for controlling bacterial diseases, the emergence of antimicrobial resistance (AMR) and its potential adverse impacts on the environment, as well as on animal and human health, necessitate a paradigm shift. Despite advancements in vaccine development and sanitary management, the search for alternative solutions (e.g., next-generation vaccines, probiotics, and prebiotics, among others) and the optimization of existing strategies are imperative to ensure the industry’s long-term sustainability. In this context, there is a pressing need to develop a specific roadmap for Chilean salmon farming to guide actions aimed at improving bacterial disease management. Such a roadmap would enable sustainable production grounded in the ecosystem approach to aquaculture, recognizing that the three pillars (i.e., economic, environmental, and social sustainability) are complementary and should not involve systematic trade-offs [10].

In Chile, SERNAPESCA regulates AMU and offers a voluntary certification program, the Optimization of Antimicrobial Use Program (PROA, Spanish acronym), which promotes AM reduction by optimizing its use and, consequently, mitigating AMR [11]. Since 2016, the specific mandatory program on antibiotic-use reporting by farmers that has been formalized, has provided Chile with a unique database, making its salmon aquaculture sector an exceptional case study at the global level [5,12].

Despite the ongoing efforts of both the government and the salmon industry, additional work is needed to address AMU and AMR effectively. To this end, recommendations exist for developing a stewardship roadmap that promotes the prudent use of AMs in Chilean salmon aquaculture. As part of this effort, the First Congress, “Sustainable Management of Aquaculture Bacterial Diseases: An Interdisciplinary Approach” was held from 20 November to 22 November 2024, in Puerto Varas, Chile. This event brought together experts, researchers, government officials, and key stakeholders from the salmon industry to collaborate by sharing expertise and providing a platform for analyzing current challenges, exchanging knowledge, and proposing innovative solutions related to disease management, environmental impact, education, and alternatives to AMU.

As a part of the Congress, a one-day workshop was held to develop a comprehensive roadmap for prudent and responsible AM stewardship in Chilean salmon aquaculture. This unique initiative adopted a multifaceted approach encompassing environmental sustainability, therapeutic alternatives, treatment efficacy, prevention strategies, and effective communication and education programs aligned with the One Health concept, combining human, animal and environmental health [13].

The workshop, which for the first time brought together key stakeholders from the public and private sectors, academia, non-governmental institutions (NGO), and civil society, was organized around four main thematic areas:

1. AMR Prevention: Strategies were discussed to better understand, detect, and disrupt the emergence of AMR in salmon aquaculture.
2. Communication and Education: Effective communication and education programs were designed to promote responsible AMU among all stakeholders within the salmon industry.
3. Therapeutic Alternatives and Efficacy: The exploration of alternative therapeutic strategies and their efficacy in controlling infectious diseases in salmon was identified as a central focus.
4. Environmental Impact Assessment: A comprehensive evaluation of the environmental consequences of AMU in aquatic ecosystems was planned.

The outcomes of these roundtables are expected to contribute to the development of comprehensive national guidelines that will support a more informed, prudent, and responsible AMU in the Chilean salmon industry. These guidelines will serve as a foundation for the formulation of public policies, the design of educational programs, and the establishment of industry best practices. This unprecedented initiative will also contribute an interdisciplinary roadmap with global relevance, applicable not only to aquaculture but also to other animal-farming sectors.

## 2. Results

### 2.1. Participants

**Figure 1:**
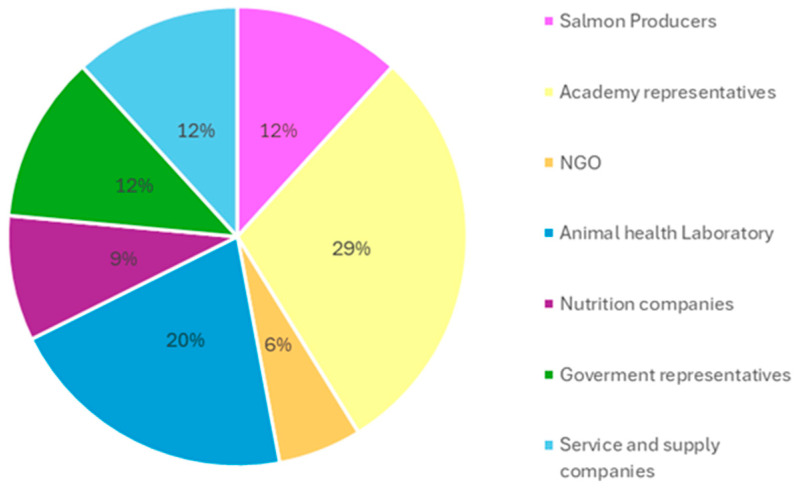
Distribution of experts who participated in the roundtables, categorized by area of expertise and professional roles.

Among the experts and professionals who attended the roundtables (Figure 1), 59% were male and 41% were female. Three countries were represented: Chile (94%), USA (3%), and Spain (3%).

### 2.2. Roundtables

Each roundtable successfully addressed its intended goals by facilitating discussion around the relevant guiding questions. A comprehensive summary of the topics, questions, and responses is presented in Table 1.

## 3. Discussion

To prevent and control infectious diseases in aquaculture, a range of fish health strategies should be effectively implemented, each targeting different stages of disease prevention. Strategies related to structural aspects of the salmon industry (i.e., primordial interventions) that underline the prevention of risk factors are typically implemented through regulations. These strategies include interventions such as defining salmon management areas, establishing minimum distances between farms, implementing biosecurity measures, and setting fallowing periods, among others. Primary interventions are implemented at the fish and farm levels and include vaccination, selective breeding for genetic resistance, feed additives, and hygiene practices, etc. Secondary interventions are related to diagnostic testing for early detection and the implementation of active surveillance [14]. Tertiary interventions, in contrast, involve strategies designed to minimize the impact of the disease, such as AMU. While AM treatments are part of the broader disease management framework, they represent a last-resort intervention and should only be applied after earlier preventive measures, spanning the primordial, primary, and secondary levels, have been effectively implemented and evaluated.

Selecting the most appropriate intervention within each strategy, and assessing its effectiveness, remains a complex challenge that requires an integrated and evidence-based approach [15]. In this context, a comprehensive understanding of AMU in aquaculture must adopt a multi-level framework that prioritizes prevention, explores alternative solutions, and ensures responsible management capable of anticipating, preventing and detecting disease early to effectively control bacteria using antibiotics.

The prevention of AMR (Section 3.1) is fundamentally based on reducing antibiotic reliance through measures such as biosecurity, vaccination, genetic selection for resistance, and improved farm management practices. Equally essential is communication and education on AMU (Section 3.2), ensuring that industry professionals, veterinarians, and other stakeholders are well-informed about responsible antibiotic stewardship, the risks associated with misuse, and the importance of an informed local community supported by communication strategies that foster understanding through clear and accessible language.

As part of secondary prevention, the development and evaluation of alternatives and therapeutic efficacy (Section 3.3) provide critical options such as probiotics, immunostimulants, and novel treatments that can reduce the need for traditional antibiotics. Finally, understanding the environmental impacts of AMU in aquaculture (Section 3.4) is crucial for assessing the long-term consequences of AM treatments, including their role in resistance development and ecosystem disruption. This reinforces the need for environmental monitoring, regulatory oversight, and the adoption of sustainable practices.

By addressing AMU across these levels of intervention, aquaculture health management can evolve toward more sustainable, effective, and responsible disease control strategies that are aligned with the principles of One Health.

### 3.1. AMR Prevention

#### 3.1.1. Understanding the Role of Aquaculture in the Development of AMR

The experts’ discussion revealed a nuanced understanding of aquaculture’s role in AMR, recognizing that it can act both as a source and a recipient of resistant bacteria. This dual role is influenced by patterns of AMU and the characteristics of the specific aquaculture system in place. Although recent risk analyses have suggested an overall low probability of AMR acquisition through the consumption of salmon filets treated during production [16], evidence of AMR has been found in sediments impacted by AMU in human medicine, raising concerns that aquaculture-related AMU may further contribute to this issue [8].

#### 3.1.2. Proposing Public Policies to Prevent the Development of AMR in Aquaculture

Experts emphasized the need for comprehensive AMR surveillance programs that adopt a One Health approach, integrating efforts across human, animal, and environmental health. These programs should be supported by both public and private funding and involve broad stakeholder participation. Key recommendations include establishing supra-institutional governance structures that emphasize coordinated stakeholder engagement, policy frameworks, and collaborative strategies. The formation of a dedicated panel of experts to monitor new research and technologies applicable to aquaculture was also proposed. Additionally, experts suggested that alternative industries could advise aquaculture regulators on new and relevant technologies that might be applied to AMU and AMR management.

In addition, Miranda, Godoy [12] suggested that Chilean salmon farms should be required to conduct AMR studies on sediments beneath cages, implement standardized protocols, and ensure that AMR monitoring data are accessible to all relevant stakeholders.

#### 3.1.3. Identifying Key Stakeholders Responsible for Preventing and/or Mitigating AMR Risks

Stakeholders across the Chilean aquaculture sector have implemented a range of preventive strategies to mitigate AMR risks, many which have proven effective in reducing AMU. These include improvements in smolt quality management, implementation of robust biosecurity measures, proper vaccine administration, early therapeutic interventions, health surveillance, and the control of coinfecting agents such as sea lice. Additional practices include the use of functional feeds and bioactive additives to improve fish welfare, optimized oxygen management, predator control, selective breeding for disease resistance, and shorter production cycles (e.g., growth periods of approximately ten months at sea for some species; between 2010 and 2014, the production cycle for *Salmo salar* decreased by four months).

Currently, Chile has several distinct AMU-reduction programs in place, each following a different approach. For instance, Yelcho is a Public–Private partnership initiative focused on accelerating vaccine implementation and reducing antibiotic use. The Pincoy Project (https://proyectopincoy.com/en/, accessed on 15 February 2025), a private initiative, addresses a wide range of production practices, including smolt selection, genetic improvement, and high-performance diets. CSARP is a collaborative initiative between the Monterey Bay Aquarium, a non-governmental organization, and the Technological Salmon Institute (INTESAL), aimed at improving management and prevention practices. PROA, on the other hand, is a voluntary certification program led by SERNAPESCA [4,5]. Recently, SERNAPESCA incorporated a new initiative, the Surveillance, Alert, and Response System to Reduce the Use of Antimicrobials (SVAR, Spanish acronym), supported by the International Center for Antimicrobial Resistance Solutions (ICARS).

In parallel, the World Organization for Animal Health (WOAH) and the Food and Agriculture Organization (FAO) have collaborated on a strategy to improve AM data collection and management. This collaboration led to the implementation of ANIMUSE, a global platform launched in 2022 to support AMU reduction efforts worldwide [17].

#### 3.1.4. Identifying Effective Strategies for Communicating AMR Risks to Aquaculture and Public Health Stakeholders

In Chile, authorization procedures, product registration, and AMU in aquaculture are strictly regulated by the Aquaculture and Livestock Authority (SAG, Spanish acronym) and enforced by various governing institutions, as reviewed by Miranda, Godoy [12] and Alvarado-Flores, Encina-Montoya [18]. Treatments may only be administered with a veterinary prescription and are almost entirely (99%) delivered as medicated feed pellets formulated by commercial feed manufacturers [12,19,20]. Other studies have shown that only 0.1–0.3% of feed is lost during normal operations when functional feed monitoring systems are used, which is favorable compared to other salmon aquaculture operations [21].

Additionally, aquaculture facilities in Chile are required to comply with environmental regulations, including emission standards for contaminants discharged into marine and continental surface waters [22], which currently do not include antibiotic discharge.

### 3.2. Communication and Education on AMU

Based on the experts’ analysis, it is essential to project how to implement the recommended changes in communication strategies related to antibiotic use and its associated risks. This could be achieved through several coordinated objectives, including:

- Developing a comprehensive project with the overarching goal of designing and implementing a targeted communication strategy.
- Establishing an interdisciplinary team that includes communication professionals to ensure that dissemination efforts are guided by robust scientific and expert knowledge.
- Identifying specific target audiences to ensure effective education and awareness-raising, as well as determining the most appropriate information channels for each audience.
- Training communication platforms, including the press and media channels, to ensure that messages are accurate, consistent, and help to prevent misinformation.

From an educational standpoint, experts noted significant gaps in veterinary and aquaculture education programs, particularly in specialized aquaculture training. Currently, this specialization is largely limited to postgraduate studies (master’s and PhD levels). The development of human capital with strong aquaculture expertise is urgently needed to address emerging challenges in the sector. Students often lack awareness of relevant public policy, as much of this knowledge remains concentrated within the industry. This is not just a local issue; Bondad-Reantaso, MacKinnon [9] also emphasized the global importance of training aquatic health professionals to improve antibiotic use management in aquaculture.

Finally, experts estimated that the process of embedding this knowledge and shifting perceptions among the various target audiences will require approximately 10 years, the time span of an entire generation, for these changes to become fully internalized and reflected in practice. Achieving this transformation will require efforts over the medium and long term.

### 3.3. Alternatives and Therapeutic Efficacy

#### Exploring Effective Alternatives and Preventive Tools Against Bacterial Infections in Aquaculture

This section aims to identify alternative treatments for bacterial diseases in aquaculture and assess their therapeutic efficacy. It also seeks to review and outline preventive measures, including natural products and vaccines, that are currently in use or under development for the control of bacterial diseases.

Bondad-Reantaso, MacKinnon [9] conducted a global review of alternative methods to AMU in aquaculture. Their study highlighted vaccines as one of the most effective tools for disease prevention in finfish, outperforming other reviewed approaches such as prebiotics, probiotics, bacteriophages, bacteriocins, medicinal plants, quorum quenching, and immunoglobulin (IgY) derived from chicken egg yolk.

From the Chilean perspective, by 2017, a total of 32 vaccines targeting *P. salmonis*, the main bacterial pathogen affecting salmon farming, were registered with the SAG [23]. Currently, 62 vaccines against bacterial pathogens are registered with the SAG [24]. However, despite availability, the demonstrated effectiveness of these vaccines remains limited, which continues to pose a challenge for disease control [23,25,26]. Some researchers suggest that maintaining optimal sanitary conditions, along with the use of environmentally friendly products, may represent more effective alternatives to AMU [8].

The Pincoy project, presented during the Congress, emphasized the importance of implementing good practices to reduce mortality caused by *P. salmonis* and to decrease AMU. Among the most impactful strategies were genetic selection, vaccination, functional feeds, and effective sea lice control.

Therapeutic efficacy against *P. salmonis* was evaluated in two studies presented at the Congress. Both demonstrated that timely intervention, specifically the prompt initiation of antibiotic treatment, significantly reduces mortality, shortens treatment duration, and, consequently, lowers AMU.

Several notable innovations in natural products were also presented. These included the use of plant extracts as antibiotic alternatives, supplementation of the intestinal microbiota, the development of new technologies for cost-effective and accurate detection of AMR, and passive immunotherapy using alpaca-derived antibodies for salmonids. All of these alternative therapies have been developed by Chilean companies in collaboration with the national salmon industry, representing a significant scientific contribution to sustainable aquaculture and fostering a specialized knowledge hub in southern Chile in relation to a sector of high economic significance.

Further research efforts in Chile have also focused on in vitro testing of plant-based extracts such as *Quillaja saponaria* (commonly known as “Quillay”) for potential activity against *P. salmonis* [27].

Lastly, it is necessary to analyze the current regulatory framework governing the coherent registration of both chemical and biological products used as natural alternatives in aquaculture to ensure that these innovative solutions can be safely and effectively integrated into practice.

### 3.4. Environmental Impacts of Antibiotic Use in Aquaculture

#### 3.4.1. Assessing the Environmental Impact of AMU in Aquatic Ecosystems

According to the expert panel, determining the precise environmental impacts of AMU in aquaculture remains challenging due to insufficient data and the difficulty of distinguishing aquaculture-related effects from other anthropogenic sources of pollution. Farías, Ibarra [5] similarly observed that significant data gaps still hinder accurate assessments of aquaculture’s impact on aquatic ecosystems worldwide. Carrizo [28] showed that the presence of antibiotics intended for human use has become much more relevant than those originating from aquaculture production. However, it is well established that the presence of antibiotics in aquatic environments can alter microbial communities surrounding aquaculture sites [4].

#### 3.4.2. Methodologies for Environmental Impact Assessments and Existing Knowledge Gaps

Experts emphasized the importance of using advanced scientific tools, particularly molecular, analytical, and kinetic techniques, to evaluate the environmental impacts of AMU, as well as to understand the dynamics and fate of these substances in aquatic ecosystems. These methods are essential for determining the occurrence of AM compounds and their effects on individual organisms, thereby advancing knowledge toward higher levels of biological organization.

Recognizing appropriate methodologies for evaluating environmental impacts in the aquatic environment requires considering factors such as technique sensitivity and available analytical capacity. The lack of mathematical models capable of predicting the presence of molecules in the environment has limited the development of effective risk-assessment methodologies. As a result, few approaches adequately reflect specific conditions for assessing the dispersal of AMs during treatments in open-net pens [29].

The sensitivity and availability of analytical techniques also play a critical role in accurately detecting and quantifying antibiotic residues in water and sediments. Despite recent advances, there remains a pressing need to strengthen analytical capacity and establish standardized methodologies to support comprehensive environmental assessments. In many cases, to address these challenges, effect-based methods have been used for the monitoring and assessment of chemical pollutants in water bodies, given the complexity of chemical mixtures present in the environment. These methods are designed to identify the potential causes of observed effects by targeting the main modes of action of free molecules found in aquatic ecosystems [30,31].

Progress in research and technology has enabled more effective monitoring and mitigation of AM contamination. Several international organizations, including the FAO, WOAH, World Health Organization (WHO), and the World Bank, have invested in developing tools, training programs, and technical guidelines to improve AMU practices and raise awareness of associated environmental risks.

The WHO has identified a list of critically important antibiotics for human health, many of which are also used in aquaculture. The FAO and WOAH have also published extensive resources and conducted workshops on AMU challenges, responsible practices, and AMR risk management [32].

#### 3.4.3. The Necessity of Conducting an Environmental Risk Assessment

An Environmental Risk Assessment (ERA) is a structured process used to estimate the likelihood of adverse effects on ecological systems, typically at the organism level, resulting from exposure to chemical substances. Its primary objective is to identify and characterize risks through integrated effect and exposure assessments, providing scientific support for decision-making aimed at preventing unacceptable harm to the environment and biodiversity [33,34]. Although ERAs can be applied across various ecological scales, progress at the local level has been limited, mainly due to the scarcity of predictive models and the limited understanding of microbial communities. This knowledge gap restricts the ability to assess the broader ecological consequences of trace levels of antibiotics, particularly their potential impacts at higher levels of biological organization [35]. To address these limitations, ERA procedures should be refined to incorporate the evaluation of microbiota-level effects based on exposure and to establish mechanistic links between microbial disruption and alterations in key biogeochemical processes.

#### 3.4.4. Identifying Regulatory Gaps and Acceptable Standards

Several key elements should be considered when developing acceptable standards and effective environmental regulations to minimize AM impacts. For example, a regulatory system of environmental assessments should include the evaluation of local exposure and effect scenarios, prioritization of substances based on their impact, estimation of load per area, and definition of maximum allowed levels based on appropriate indicators. Regulations on maximum density per center and per ecosystem should also be implemented and gradually reduced to limit AMU.

Strengthening regulations for the control, use, and final disposal of veterinary and human pharmaceuticals with an environmental focus is essential. Moreover, academia should be incentivized to expand research on the environmental pharmacokinetics of residues and their ecological effects, ensuring that knowledge is shared among stakeholders across academia, the private sector, and the public sector.

A recent publication by Toonen, Bush [2] provided a comprehensive review of sustainable governance in the global aquaculture sector. The study identified weak civic engagement and limited coordination across many countries. However, Chile stood out for its strong coordination in disease prevention, largely driven by social pressure. Despite this progress, challenges persist. While Chilean regulation supports disease management, it still struggles to provide effective and long-term solutions. Environmental challenges remain insufficiently addressed by existing legal frameworks. The authors emphasized the need for improved governance and stronger collaboration between the government, industry, and civil society organizations to ensure more resilient and sustainable disease and environmental management in aquaculture.

### 3.5. The Roadmap

**Figure 2:**
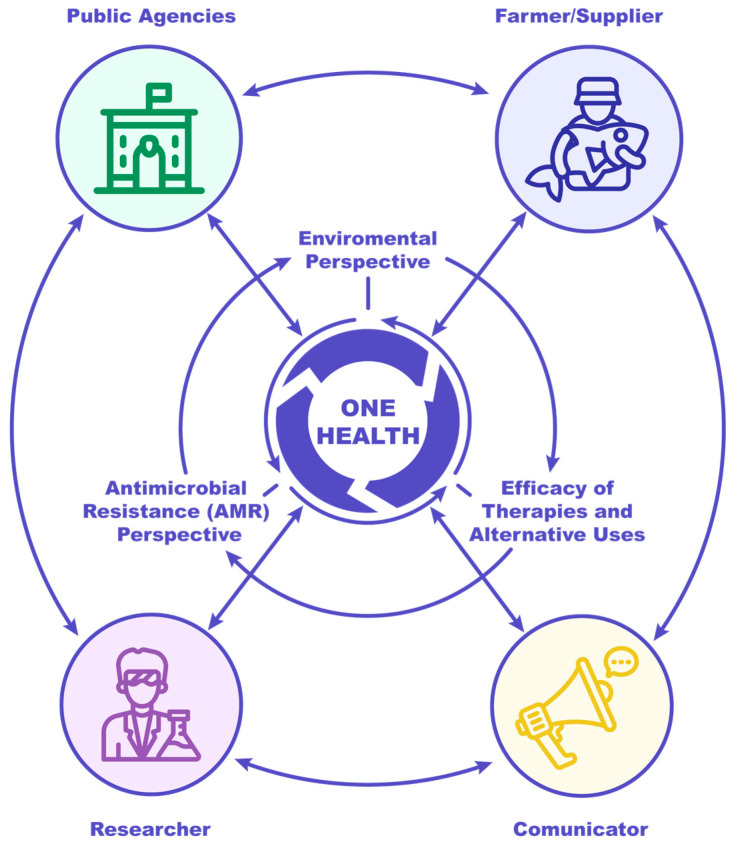
Elements of the roadmap emphasizing interconnection (actions) and multidisciplinary approach (stakeholders) under the One Health framework. Arrows indicate interaction between different actors and stakeholders under the One Health framework.

This roadmap aims to improve understanding, detection, and disruption of AMR emergence in farmed salmon under a One Health framework (Figure 2). It seeks to reduce, replace, optimize, improve access to, and innovate AMU by supporting multisectoral, collaborative, and interdisciplinary research that will improve understandings of and provide new opportunities for AMR prevention and combat. Achieving a sustainable and responsible future for aquaculture requires a well-defined roadmap. While a comprehensive roadmap specifically tailored for Chilean salmon aquaculture is presented in an associated policy brief (unpublished work), this manuscript presents a broader set of globally relevant recommendations aimed at guiding actions toward sustainable and responsible aquaculture practices. Summarized in Table 2 are recommendations targeting public agencies, salmon producers, researchers, and communication professionals, addressing environmental issues, AMR, and the efficacy of therapies and alternatives to AMU. Meanwhile, Table 3 focuses specifically on communicators, providing guidance on addressing communication challenges and capacity-building needs related to AMU in Chile’s salmon industry.

### 3.6. Challenges and Limitations

The development of a comprehensive roadmap for sustainable antimicrobial use (AMU) in Chilean salmon farming faces substantial implementation challenges in four critical areas:

1. Data and knowledge gaps on environmental impacts: Insufficient data make it difficult to accurately quantify the environmental consequences of AMU and to distinguish aquaculture impacts from other pollution sources.

2. Regulatory and governance hurdles: Current legal frameworks are weak, with environmental challenges inadequately addressed. Crucially, current emission standards for contaminants do not include antibiotic discharge. Implementing effective, long-term solutions is challenging, and establishing the necessary supra-institutional governance for integrated “One Health” surveillance presents significant logistical complexities.

3. Industry and economic constraints: The sector’s high dependence on AMU persists. Adoption of alternatives is slowed by the need for rigorous in-field validation and the potential for increased production costs.

4. Educational limitations: Achieving a generational shift in practices requires a sustained effort over roughly ten years to address gaps in specialized veterinary and aquaculture training and to integrate new knowledge across all stakeholders.

## 4. Materials and Methods

To achieve the stated objectives, a total of 41 experts from the productive sector, academia, and government regulatory agencies were invited to share their ideas, opinions, and perspectives in a series of roundtable discussions organized according to their areas of expertise on the four main proposed topics.

### 4.1. Participants

**Figure 3:**
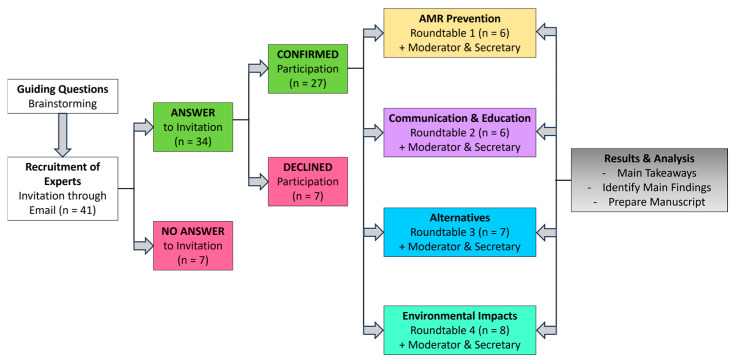
Methodological framework used to design and organize roundtable discussions.

The selection criteria for experts were based on their field, years of experience in the selected topic, aquaculture background, involvement in governance and decision-making, affiliations with regulatory agencies, business partnerships, academia, research institutions, and the organizing team. A total of 41 experts were contacted via email and invited to join a specific roundtable according to their area of expertise (Figure 3).

Participation was voluntary, and each expert was fully informed of the purpose and scope of the activity, ensuring their autonomy and right to withdraw from the study at any time. No personal, confidential, or sensitive data were collected; instead, the selection prioritized their level of expertise in the topics under discussion.

Each expert who agreed to participate signed a consent form prior to the meeting at the Congress, outlining the terms of participation, and completed a questionnaire containing up to ten questions relevant to their area of expertise.

### 4.2. Roundtables

The meeting was held in person on 22 November 2024, and lasted for two hours. Each roundtable was formed with consideration to gender equality and the inclusion of expert representatives from each sector, as well as including representatives from the organizing team, who moderated and guided the work. All moderators possessed extensive knowledge of the session’s topic.

The discussions followed a structured format that began with a set of pre-developed questions (Table 1), followed by an open discussion led by both a moderator and a designated secretary. The moderator and secretary facilitated the dialog and ensured the active participation of all attendees.

ROUNDTABLE 1—AMR PREVENTION Goals:

1. To describe the role of aquaculture in the development of AMR.
2. To propose public policies aimed at preventing the emergence and spread of AMR in aquaculture.
3. To identify key stakeholders responsible for preventing and/or mitigating AMR-related risks.
4. To identify effective methods for communicating AMR risk to aquaculture stakeholders and public health authorities.

ROUNDTABLE 2—COMMUNICATION AND EDUCATION OF AMU Goals:

1. To identify effective mechanisms for disseminating information within the aquaculture industry and to the broader community.
2. To propose key topics to be included in veterinary medicine curricula.
3. To identify available technologies that can be leveraged for communication and education on AMU.

ROUNDTABLE 3—ALTERNATIVES AND THERAPEUTIC EFFICACY Goals:

1. To identify alternative treatments for bacterial diseases in aquaculture and evaluate their efficacy.
2. To review and describe preventive measures, such as natural products and vaccines, currently in use or under development for the control of bacterial diseases.
3. To propose methodologies for efficacy testing.
4. To analyze the current regulatory framework for the registration of pharmaceutical and biological products.

ROUNDTABLE 4—ENVIRONMENTAL IMPACTS OF AMU IN AQUACULTURE Goals:

1. To determine the potential impacts of AMU on the aquatic environment.
2. To identify methodologies for evaluating environmental impacts in the aquatic environment, including, for example, technique sensitivity and available analytical capacity.
3. To identify existing knowledge gaps.
4. To discuss the need for conducting an environmental risk assessment.
5. To identify regulatory gaps and define acceptable standards.

## 5. Conclusions

AMR Prevention: AMR must be recognized not only as a public health concern but also as an environmental issue. Addressing AMR from an ecological perspective is essential to anticipate long-term consequences, evaluate the impact of current practices, and develop sustainable solutions before these are enforced by regulatory mandates or triggered by environmental crises. Proactive action, rather than reactive regulation, should guide efforts toward responsible AMU. In this context, international collaboration and the implementation of preventive policies will be critical to effectively manage and mitigate the growing threat of AMR in aquaculture and beyond.

Communication and Education: For the first time, AMU in the Chilean salmon industry has been addressed from a communication and education perspective. Experts emphasized that effective communication, combined with comprehensive education and professional training, is essential to promote responsible practices and support the transition toward sustainable aquaculture. They projected that achieving meaningful changes in awareness, perception, and behavior across stakeholders will require a sustained effort over approximately ten years, reflecting the generational scale of transformation needed.

Therapeutic Alternatives and Efficacy: While vaccines are widely regarded as the most effective strategy for preventing bacterial diseases in aquaculture, limited efficacy against *P. salmonis* in Chile remains a significant challenge. In response, Chilean researchers have actively pursued the development of alternative therapies. These innovations, ranging from natural products to novel immunological approaches, represent a substantial scientific contribution to the pursuit of more sustainable and resilient aquaculture practices.

Environmental Impact Assessment: Environmental concerns related to AMU extend beyond the aquaculture sector and intersect with broader human health and ecological systems. Addressing these challenges requires a comprehensive approach that considers external aggravating factors, such as the documented links between pharmaceutical residues and the development of AMR. To advance understanding and support evidence-based science, it is essential to incentivize academic research focused on the environmental dynamics and fate of AM residues and their effects at higher biological levels, reinforcing risk assessment procedures. This knowledge must then be effectively communicated across sectors, including academia, industry, and government, to inform responsible decision-making and foster coordinated action.

Roadmap: The insights presented in this manuscript, drawn from the Chilean salmon farming experience concerning AMU in aquaculture, make a significant contribution to the industry’s journey toward sustainability. Although several areas still require further development, considerable progress has been achieved. This case study provides a strong foundation for building a roadmap and formulating globally applicable recommendations for stakeholders involved in aquaculture industries worldwide. Finally, international collaboration is crucial to rapidly translating the key lessons and insights from the Chilean experience into globally applicable standards.

## Figures and Tables

**Table 1:** Summary of key findings from the open-discussion roundtables. AM: Antimicrobial; AMR: Antimicrobial Resistance; AMU: Antimicrobial Use.

ROUNDTABLE 1—AMR PREVENTION	
Question	Comments
Is aquaculture a source or receptor of AMR? Are there tools to demonstrate AMR and/or how can it be measured?	Aquaculture plays a dual role as both a source and a recipient of AMR, depending on antibiotic use and aquaculture type.
Have public–private programs to reduce AMU and the risk of AMR been effective, or what should be changed?	Chile has several initiatives that have had success in reducing AMU. However, these have not effectively tackled the reduction in AMR
What should an AMR monitoring program be like in aquaculture?	AMR surveillance programs should be comprehensive, adopting a “One Health” approach, and be supported by both public and private financing and participation, should feature supra-institutional governance and be guided by an expert advisory committee.
If more knowledge about AMR in aquatic environments is required, how long could it take and what costs could it entail?	The salmon industry has implemented a range of effective strategies to reduce AMU, including smolt quality management, biosecurity measures, proper vaccine use, early treatments, health surveillance, *sea lice* control, fish-welfare bioactive additives, functional diets, oxygen management, predator control, genetic improvements, and shorter time spent at sea.
What are the minimum elements that a regulation should have for responsible AMU?	Chilean regulations promotes the prudent and responsible use of AM, through diagnostic requirements, veterinary prescriptions, and the prohibition of growth promoters. However, there is a need to update the registry of authorized products to include treatment efficacy data and to implement positive incentives, rather than just restrictions or obligations.
**ROUNDTABLE 2—COMMUNICATION and EDUCATION OF AM USE**	
How can the training of human capital in aquaculture be improved? What gaps in professional training in animal health exist or should be improved for better management of AMU?	Veterinary training provides adequate knowledge for professionals to thrive in the aquaculture industry. However, as with any professional, continuous education and specialization are essential for ongoing development.
How should stakeholders and communities be informed about aspects related to the AMU in aquaculture? What technological or communication tools can be used to communicate about AMU at different levels?	To take action at multiple levels to raise awareness. Education on AMU plays a key role. The information must be delivered with a clear, coherent, and targeted message tailored to each audience, using the appropriate tools.
What are the aspects of greatest concern regarding management, use, and impacts from the point of view of the salmon value chain?	Increased production costs, environmental impact, AMR, repercussions on human health, consumer perception, and effects on the industry’s image.
**ROUNDTABLE 3—ALTERNATIVES AND THERAPEUTIC EFFICACY**	
What alternatives to AMU as a treatment for fish infections are foreseen in the short term?	Standardized testing of functional diets is urgently needed. Although new vaccines show promise, they are not expected to be available in the near future. Maintaining animal welfare must remain a top priority.
How should these tools be made effective and available to fish farmers?	Feeding strategies during treatment should be further studied. Experimental marine centers could play a key role in advancing this research.
What practices have been shown to reduce AMs consumption without compromising mortality?	Early treatment is crucial for maintaining the health of fish living with infected individuals and for reducing the risk of death in the infected fish. Factors such as predator control and water quality also play significant roles. Freshwater aquaculture is equally important but faces unique challenges, particularly due to climate change.
How could AM dosages be optimized in a treatment?	Collaboration between pharmaceutical companies and feed mills. Optimizing and validating therapeutic doses is essential to ensure the effectiveness of treatments. Decisions regarding the use of doses should be guided by pharmacovigilance data to ensure both safety and efficacy. Update Minimal Inhibitory Concentration values for AMs against P. salmonis, considering factors such as virulence. Emphasizing the importance of consistent culture conditions for ensuring treatment success.
What are the necessary elements for successful treatment?	Better tools are needed to support more informed decision-making regarding treatments. Detailed records and ongoing monitoring are essential for tracking treatment effectiveness. The overall health of the fish, particularly gut health
**ROUNDTABLE 4 —ENVIRONMENTAL IMPACTS OF ANTIMICROBIAL** ** USE IN AQUACULTURE**	
What are the specific environmental impacts of AMU in freshwater and marine aquaculture on aquatic ecosystems?	Potential negative effects of AMU in aquaculture on the overall health and balance of aquatic ecosystems.
What are the most suitable methods and tools for evaluating the environmental impacts of AMU in aquaculture?	There is insufficient data to quantify the exact impact of aquaculture and other sources. Molecular, analytical, and kinetic tools are crucial for studying these impacts. Scientific methods and techniques used to study biological processes at a molecular level are essential for understanding the effects of AMs on organisms and ecosystems.
What additional information or studies are needed to have a more complete understanding of these long-term impacts?	The importance of bioassays is in investigating how microorganisms’ function within an ecosystem and how these functions are affected by factors such as AMU. Emphasis should be placed on real-world conditions, the passage of time, and regional differences when studying the impacts of AM. The concept of “ecological cancerization” refers to the long-term, irreversible damage that can occur to an ecosystem.
Considering the environmental impacts of AMU in aquaculture, what key elements should an effective environmental regulation include to minimize risks?	Environmental regulations regarding AMU should be regularly updated to incorporate new scientific findings and improve our understanding of the impact of these compounds.

**Table 2:** Roadmap and strategic recommendations for priority areas derived from an expert workshop addressing environmental issues, antimicrobial resistance (AMR), and the efficacy of therapies and alternative uses of antibiotics. Actions are prioritized and categorized by the primary responsible stakeholder (Public Agencies, Farmer/Supplier, Researcher, Communicator) across three critical domains.

Domain	Stakeholder	Prioritized Action
**ENVIRONMENTAL PERSPECTIVE**	**Public Agencies**	Establish an integrated “One Health” governance framework for environmental health policy.
		Mandate Environmental Risk Assessments (ERAs) within AM product authorization processes, ensuring transparency and reliable reporting.
		Delineate and implement ecosystemic measurement protocols that account for the spatial and temporal variability of xenobiotics, encouraging collaboration and continuous improvements.
	**Farmer/Supplier**	Implement protocols for minimizing AM environmental dissemination and ensure preparedness for ERA compliance.
	**Researcher**	Develop and validate rigorous ecological impact assessment methodologies specific to intensive aquaculture systems.
		Quantify the cumulative environmental effects of AMU across relevant spatial and temporal scales.
	**Communicator**	Disseminate information consistently to promote the intersectoral “One Health” paradigm.
**ANTIMICROBIAL RESISTANCE (AMR) PERSPECTIVE**	**Public Agencies**	Mandates and resources integrated into “One Health” AMR surveillance programs.
		Modify legislation to explicitly differentiate aquaculture as both a source and recipient of AMR determinants.
		Mandate the incorporation of quantifiable efficacy metrics (e.g., decrease in resistance prevalence) into AMR reduction initiatives.
	**Farmer/Supplier**	Ensure active participation and transparent data submission to “One Health” AMR surveillance initiatives.
		Implement systematic monitoring and timely reporting of operational AMR prevalence and antibiotic consumption data.
	**Researcher**	Refine and integrate methodologies to accurately distinguish AMR origin and assess potential public health relevance.
		Design and validate objective efficacy indicators for assessing the performance of AMR mitigation strategies.
	**Communicator**	Synthesize and communicate complex AMR data effectively, differentiating between sources for public understanding.
**EFFICACY OF THERAPIES AND ALTERNATIVE USES**		Establish positive financial and regulatory incentives to promote the development and adoption of non-antibiotic prophylactic and therapeutic agents.
	**Public Agencies**	Resource and establish on-site demonstration and experimental facilities for real-world validation of innovative strategies.
		Require the submission of robust, validated efficacy data for the renewal and registration of veterinary medicinal products.
	**Farmer/Supplier**	Integrate and enforce evidence-based management practices proven to minimize antibiotic consumption (e.g., biosecurity optimization).
		Proactively evaluate and adopt validated non-AM alternatives (e.g., functional feeds, contemporary vaccines).
		Optimize the inclusion and delivery mechanisms of veterinary therapeutics via feed to ensure appropriate dosage administration.
	**Researcher**	Conduct rigorous, real-world field validation studies on the efficacy of non-antibiotic interventions.
		Prioritize research on AM population pharmacokinetics and the optimization of therapeutic dosing regimens.
	**Communicator**	Facilitate the knowledge transfer of scientific efficacy data and alternative therapeutic options to stakeholders.

**Table 3:** Roadmap and recommendations for communicators, derived from an expert workshop focused on communication challenges and capacity building related to antimicrobial use (AMU) in the Chilean salmon industry.

Domain	Prioritized Action
**1.** **Strategic Communication Plan Development**	**Develop and implement a comprehensive communication strategy.** Initiate a project to create and roll out a detailed communication strategy that involves forming an interdisciplinary team with communication specialists and subject-matter experts to craft messaging based on expert insights. Finally, identify specific target audiences for practical education and awareness.
**2.** **Targeted Messaging and Channel Selection**	**Identify consistent distribution channels** for information that aligns with each target audience. Messages should be coherent, clear, and focused, utilizing appropriate tools to effectively transmit AMU information in aquaculture and its risks.
	**Highlight the critical importance of collaborative work** between industry and universities. This partnership is key to understanding industry needs and enabling universities to contribute solutions, both in professional training and scientific research.
	**Strengthen veterinary education in aquaculture** by advocating for and supporting the development of specialized modules or programs in aquaculture. Emphasize the importance of continuous education and specialization (i.e., diplomas, MSc, PhD) for veterinary professionals already in the field.
**3.** **Long-Term Perception Change Strategy**	**Develop a continuous communication roadmap** with long-term goals and consistent messaging to achieve a generational shift.
	**Establish mentorship programs** that connect experienced professionals with new graduates to facilitate knowledge transfer and integration into the industry, with best practices over the long term.
**4.** **Campaign Evaluation**	**Design communication campaigns** with specific evaluation indicators to measure effectiveness and impact.

## Data Availability

No new data were created or analyzed in this study. Data sharing is not applicable to this article.
